# Raising Awareness of False Positive Newborn Screening Results Arising from Pivalate-Containing Creams and Antibiotics in Europe When Screening for Isovaleric Acidaemia

**DOI:** 10.3390/ijns4010008

**Published:** 2018-02-10

**Authors:** James R. Bonham, Rachel S. Carling, Martin Lindner, Leifur Franzson, Rolf Zetterstrom, Francois Boemer, Roberto Cerone, Francois Eyskens, Laura Vilarinho, David M. Hougaard, Peter C.J.I. Schielen

**Affiliations:** 1 Sheffield Children’s NHS FT, Sheffield, S10 2TH, UK; 2 South East Thames Regional Newborn Screening Laboratory, Biochemical Sciences, Viapath, Guy’s & St. Thomas’ NHS FT, London, SE1 7EH, UK; 3 Division for Neuropediatrics and prevention, Department of General Pediatrics, University of Frankfurt/Main, 60590 Frankfurt, Germany; 4 Department of Genetics and Molecular Medicine, University of Iceland, Landspitali, 101 Reykjavik, Iceland; 5 Centre for Inherited Metabolic Diseases, Karolinska University Hospital and Department of Molecular Medicine and Surgery, Karolinska Institutet, SE-17176 Stockholm, Sweden; 6 Biochemical Genetics Laboratory, Department of Human Genetics, CHU Sart-Tilman, University of Liège, 4000 Liège, Belgium; 7 Department of Pediatrics, Giannina Gaslini Institute, Largo Gaslini 5, 16147 Genoa, Italy; 8 PCMA vzw Centre for Inherited Metabolic Disorders in Children, Antwerp University Hospital UZA, 2650 Antwerpen-Edegem, Belgium; 9 Newborn Screening, Metabolism & Genetics Unit, Human Genetics Department, National Institute of Health Dr Ricardo Jorge, 4049-019 Porto, Portugal; 10 Danish Center for Neonatal Screening, Director Department for Congenital Disorders, Statens Serum Institut, 2300 Copenhagen Denmark; 11 Reference Laboratory for Neonatal Screening, Centre for Health Protection, National Institute for Public Health and the Environment (RIVM), 3720 BA Bilthoven, The Netherlands

**Keywords:** pivalate, pivoloyl, antibiotic, moisturing cream, C5 acylcarnitine, isovaleric acidaemia, IVA interference tandem mass spectrometry

## Abstract

While the early and asymptomatic recognition of treatable conditions offered by newborn screening confers clear health benefits for the affected child, the clinical referral of patients with screen positive results can cause significant harm for some families. The use of pivalate-containing antibiotics and more recently the inclusion of neopentanoate as a component within moisturising creams used as nipple balms by nursing mothers can result in a significant number of false positive results when screening for isovaleric acidaemia (IVA) by measuring C5 acylcarnitine. A recent survey conducted within centres from nine countries indicated that this form of contamination had been or was a significant confounding factor in the detection of IVA in seven of the nine who responded. In three of these seven the prominent cause was believed to derive from the use of moisturising creams and in another three from antibiotics containing pivalate; one country reported that the cause was mixed. As a result, four of these seven centres routinely perform second tier testing to resolve C5 isobars when an initial C5 result is elevated, and a fifth is considering making this change within their national programme. The use of creams containing neopentanoate by nursing mothers and evolving patterns in the prescription of pivalate-containing antibiotics during pregnancy require those involved in the design and operation of newborn screening programmes used to detect IVA and the doctors who receive clinical referrals from these programmes to maintain an awareness of the potential impact of this form of interference on patient results.

## 1. Introduction

Isovaleric acidaemia (IVA) is an autosomal recessive disorder of leucine metabolism. In the acute neonatal form, patients are typically well at birth and present during the first two weeks of life with poor feeding, vomiting, lethargy, seizures, and progressive coma. While newborn screening has enabled the pre-symptomatic identification of IVA, prompt and appropriate treatment is required to ensure that the baby is protected and, as a result, rapid referral and assessment is advocated in screen positive cases.

Unfortunately, the positive predictive value of a raised C5 acylcarnitine result, upon which many programmes depend, may be quite low [1] and the resulting false positive (FP) cases can have a serious long-term impact with an increased parental stress index score, a significant and lasting increase in parental anxiety, and an increase in hospital stays of the screen positive children [2]. A significant contribution to FP screening results for IVA in some countries may arise from pivaloylcarnitine, a compound isobaric to, and interfering with the measurement of, C5 acylcarnitine, the dedicated screening parameter for IVA [3]. Pivaloylcarnitine can be present in blood due to maternal use of pivalic ester pro-drugs or pivalic acid derivatives used as emollients in some nipple creams. Common examples of such drugs include Pivmecillinam, the pivaloyloxymethyl ester of Mecillinam, and Pivampicillin, the pivaloylmethyl ester of Ampicillin. In 2014, Boemer at al. [4] reported 50 FP IVA results detected in Belgium during an 18-month period, which were subsequently investigated and found to be due to the use of a nipple fissure unguent containing pivalic acid derivatives. Similarly, following the introduction of screening for IVA in 2012 in the UK, between 2012–2016, 24 FP and 16 true positive (TP) cases were reported, with 16 out of 24 of these FP cases attributable to pivaloyl carnitine in the blood spot sample (this report; see also [5]). A recently published false positive case attributed to the use of the Neutrophil Elastase Inhibitor Sivelestat [6] in this respect is also of note.

These findings prompted an informal survey among European countries to determine the extent of interference from pivoloyl carnitine in the detection of IVA and the steps being taken to avoid the referral of FP results.

## 2. Materials and Methods

An email survey was developed and circulated to contact persons of neonatal screening programmes in 17 European countries. The questions included: (1) whether pivalate-containing medications contributed to FP results when screening for IVA; (2) if so, whether the contribution was suspected to arise mainly from the use of moisturising creams or antibiotics; (3) whether second tier testing was in place to identify pivaloyl carnitine in the blood spot sample prior to potential clinical referral; and (4) whether testing to identify pivaloyl carnitine was routinely available subsequent to clinical referral if this was indicated.

## 3. Results

Responses were received from centres in nine countries and these are summarised in Table 1. Seven of the nine countries represented considered that pivalate within dried blood spots had been or was a significant confounding factor in the detection of IVA by newborn screening using C5 acylcarnitine alone as an indicator. Three of the seven who reported that pivalate had been or was an issue considered that this most commonly derived from maternal use of moisturising creams containing neopentanoate, while three of the seven reporting pivalate interference considered that the most common source was antibiotic use.

Four of the seven respondents had instituted the resolution of C5 isobars by liquid chromatography–mass spectrometry as a second tier test to avoid unnecessary clinical referral, and one was considering this as national policy developed.

Table 2 presents an overview of 25 FP in IVA screening in the UK, with the percentage of C5 isobaric components and information of antibiotic or use of moisturising cream, if available. The C5 concentration shows that antibiotics or cream can elevate C5 to between 1.9 and 4.4 µmol/L. Some of the FP IVA screening results with very high C5 results but without additional information (C5 range 1.0–5.8 µmol/L) also showed a pivaloyl fraction reminiscent of cream or antibiotics use.

## 4. Discussion

There is convincing evidence that the early and asymptomatic detection of inherited metabolic disorders by newborn screening can offer very significant health benefits for the affected child and the family. Nevertheless, it is also well recognised that FP results reported by national screening programmes can have a significant negative impact on families and in some cases these effects may endure for many years, resulting in increased parental anxiety and an associated increase in the frequency of hospital attendances reflecting this anxiety. It is likely that both the way in which clinical referrals are managed and conducted and the receipt of the result itself may contribute to these lasting effects.

Thus, it is clearly a benefit if FP results can be avoided and the positive predictive value of testing improved. Identifying and characterising known or suspected interferences more carefully, thus avoiding an unnecessary response to a screen positive result, contributes to this improved performance. The contribution made by pivaloyl carnitine to raised C5 acylcarnitine results when screening for IVA has been known for many years. This was clearly appreciated and dealt with in countries such as Denmark, where the use of pivalate-containing antibiotics is widespread. It seems likely that this has been less well appreciated in some countries where such use is less common. There is also some evidence that in some countries, such as the UK, the pattern of pivalate-containing antibiotics use may be changing and, in general, increasing.

More recently, the inclusion of neopentanoate as an emollient in some moisturising creams used as nipple balms by nursing mothers may also be under-recognised, and may result in a significant number of FP results when screening for isovaleric acidaemia. Sixteen cases from the UK (Table 2) and 50 cases in an 18-month period in Belgium have been reported, as well as cases in other countries (see Table 3). The data of Table 2 and Table 3 together show that C5 concentrations artificially elevated by creams or antibiotics are similar to those observed in mild or severe cases of IVA.

This survey’s results indicate that a number of countries have introduced second tier testing for C5 isobars when the initial C5 is raised [5,7]. A second tier test (as used in Denmark) was also introduced in Sweden. It began mid-November 2010 and has prevented all IVA FP screening results. Nevertheless, not all countries have implemented additional testing. It may be that in those countries where this is not routinely performed, this form of contamination is not a problem. However, clinicians receiving a referral on behalf of an asymptomatic baby with a raised C5 would be well advised to take a detailed medical history as well as exclude the use of these balms and creams. If doubt remains, an C5 isobar analysis on the original blood spot is advised. Finally, if a significant number of FP results appear to be arising from this cause then a change in policy to include appropriate second tier testing prior to referral may be indicated. The second tier assay would typically take two to three hours to perform and the delay, provided that referral could be made on the same day as the initial screen positive result was obtained, would be unlikely to adversely affect the clinical outcome in true positive cases. Adopting this approach would avoid the stress associated with false positive results and offer a significant benefit to the families who would otherwise have been affected.

Finally, it is interesting to note that in Belgium, moisturising creams containing neopentanoate have been withdrawn from distribution in maternity wards due to the risk of generating FP results in national newborn screening programmes.

Awareness of these cases should be disseminated, which is why we invite readers to report such cases to the first author of this paper, to enable an update in the future.

## Figures and Tables

**Table 1:** Summary of results from a survey in centres from nine European countries.

Centre	Country	Do Pivalate-Containing Medications Contribute to False Positive (FP) Diagnoses in Your Programme?	Is This Predominantly from Creams?	Is This Predominantly from Antibiotic Medication?	Do You Undertake c5 Isobar Analysis before Patient Referral to Reduce FPs as Part of the Screening Protocol?	Do You Undertake c5 Isobar Analysis after Patient Referral to Confirm Pivalate?	Other Comments
Sheffield and London	United Kingdom	Yes, approximately 2/3 of FP result from pivalate	Mixed	Mixed	Not at present but it is being considered	Yes, where this is suspected	None
Bilthoven -RIVM	Netherlands	None reported	Not applicable	Not applicable	No	No	None
Hessen	Germany	None reported	Not applicable	Not applicable	No	No	No
Reykjavík	Iceland	Yes (C5 levels occasionally are detected; however, they do not contribute to FP (see this table under ‘Other Comments’))	No (midwives advise the use of an Icelandic ointment)	Yes (Selexid)	No	If necessary (but only once in 10 years of practice)	Prior to isobaric analysis, the newborn screening entre checks for drug intake/use in the National Drug Database/Registry and the medical history of the mother prior to birth
Stockholm	Sweden	No, due to second tier testing	Not that we are aware of	Yes (Pivmecillinam)	Yes	-	Introduced a second tier test very soon after the introduction of screening for isovaleric acidemia
Liege and Antwerp	Belgium	Yes, a few years ago severe drawbacks in newborn screening for IVA condition, with very high rates of FP results related to the use of neopentanoate-containing moisturising cream in mothers	Yes (Mustela)	No pivalate-containing antibiotics are commercialised in Belgium	Yes		In Belgium the ointment has been removed from distribution in maternity wards based on the high FP screening rate of C5. The FP rate has since diminished significantly
Porto	Portugal	Prior to second tier testing, yes	Yes (Mustela) (confirmation of use is sought by asking the mother)	No pivalate-containing antibiotic are commercialised in Portugal	Yes	No	Starting in 2011, a yearly 29, 33, 59, 35, and 36 cases were identified. The number of cases 2016 was 5, as in 2016 a second tier test was conducted before referral
Genova	Italy	Yes	Yes (Mustela; use has diminished significantly)	No	No	No	
Copenhagen	Denmark	Yes		Predominantly antibiotic medication	Yes	n/a	The use of pivaloyl-containing antibiotics in Denmark is so massive that we have only been able to screen for IVA by implementing a second tier liquid chromatography – mass spectrometry analysis

Note: The authors feel that, since this paper is meant to raise awareness, it is appropriate to provide the commercial names of products when available. It should be kept in mind, however, that neopentanoate derivates are very widely used in the cosmetic industry as emollients and that only the use of these products within the post-partum period are associated with the analytical problems mentioned in this paper. FP: false positive.

**Table 2:** Clinically confirmed false positive screening results for isovaleric acidemia from English newborn screening blood spot programme 2012–2017.

Initial C5 on Screen (µmol/L)	% of Total C5 Isobars			Additional Information	
	Pivaloyl	Methylbutyryl	Isovaleryl	Valeryl	
	**Additional information available**				
4.4	100	0	0	0	Maternal antibiotics
4.1	98	1	1	0	Lanolin nipple cream
**3.6**	95	3	2	0	Maternal antibiotics
**3.6**	97	2	1	0	Maternal pivampicillin
**2.3**	98	0	2	0	Breast-fed; Mum using Mustela cream
**2.1**	2	91	0	9	Maternal pivmecillinam
**1.9**	97	2	1	0	Maternal antibiotics
**1.9**	97	2	1	0	Maternal pivampicillin
**1.3**	-	-	-		No sample for analysis of isobars. Short/branched chain acyl-CoA dehydrogenase deficiency deficiency.
**1.2**	0	11	89	0	Breast-fed
	**No additional information available**				
**5.8**	100	0	0	0	
**5.4**	100	0	0	0	
**3**	98	0	2	0	
**2.3**	98	0	2	0	
**2.1**	90	6	4	0	
**2**	0	47	49	3	
**1.5**	94	2	4	0	
**1.4**	98	4	3	0	
**1.3**	0	6	94	0	
**1.3**	94	3	3	0	
**1.3**	0	8	92	0	
**1.1**	86	3	11	0	
**1.1**	0	13	87	0	
**1**	0	7	93	0	
**1**	0	7	93	0	

**Table 3:** C5 concentrations in newborn dried blood spot screening.

Condition	n	C5 Conc µM (Median)	Reference	Remark
Isovaleric Acidaemia (IVA) mild/intermediate	11	0.8–4.8 (4)	1	Biochemical classification (following organic acid analysis) c.932C_T (p.A282V) mutation
IVA (mild)	6	3.7–4.39	8	All presumably mild. No genetic information. “One patient (IVA-3) was symptomatic in the first days of life but had no symptoms thereafter (19-month follow-up). None of the other patients had any metabolic derangements or symptoms so far.”
IVA severe	11	8.0–22.1 (14.4)	1	Biochemical classification (OA?)
Silevestat	2	4.49; 1.09	6	
Nipple ointment	50	1–5.2	4	
Maternal antibiotic	1	7.9	3	

## References

[1] Ensenauer R., Fingerhut R., Maler E.M., Polanetz R., Olgemöller B., Röschinger W., Muntau A.C. (2011). Newborn Screening for Isovaleric Acidemia Using Tandem Mass Spectrometry: Data from 1.6 Million Newborns. Clin. Chem..

[2] Hewlett J., Waisbren S.E. (2006). A review of the psychosocial effects of false-positive results on parents and current communication practices in newborn screening. J. Inherit. Metab. Dis..

[3] Abdenur J.E., Chamoles N.A., Guinle A.E., Schenone A.B., Fuertes A.N.J. (1998). Diagnosis of isovaleric acidaemia by tandem mass spectrometry: False positive result due to pivaloylcarnitine in a newborn screening programme. J. Inherit. Metab. Dis..

[4] Boemer F., Schoos R., de Halleux V., Kalenga M., Debray F.G. (2014). Surprising causes of C5-carnitine false positive results in newborn screening. Mol. Genet. Metab..

[5] Carling R.S., Burden D., Hutton I., Randle R., John K., Bonham J.R. (2017). Introduction of a Simple Second Tier Screening Test for C5 Isobars in Dried Blood Spots: Reducing the False Positive Rate for Isovaleric Acidaemia in Expanded Newborn Screening. J. Inherit. Metab. Dis..

[6] Yamada K., Kobayashi H., Bo R., Takahashi T., Hasegawa Y., Nakamura M., Ishige N., Yamaguchi S. (2015). Elevation of pivaloylcarnitine by sivelestat sodium in two children. Mol. Genet. Metab..

[7] Minkler P.E., Stoll M.S.K., Ingalls S.T., Hoppel C.L. (2017). Selective and accurate C5 acylcarnitine quantitation by UHPLC-MS/MS: Distinguishing true isovaleric acidemia from pivalate derived interference. J. Chromatogr. B Anal. Technol. Biomed. Life Sci..

